# Surrogate metamodels from digital image correlation for testing high-performance composite vessels

**DOI:** 10.1016/j.heliyon.2024.e29525

**Published:** 2024-04-12

**Authors:** Javier Pisonero, Manuel Rodríguez-Martín, Jose G. Fueyo, Diego González-Aguilera, Roberto García-Martín

**Affiliations:** a Department of Cartographic and Land Engineering, Higher Polytechnic School of Ávila, Universidad de Salamanca, 05003, Ávila, Spain; b Department of Mechanical Engineering, Higher Polytechnic School of Zamora, Universidad de Salamanca, 49022, Zamora, Spain

**Keywords:** Composites, Vessels, Digital image correlation (DIC), Finite element method (FEM), Testing, Reliability engineering

## Abstract

In this work, a workflow has been developed for the generation of surrogate metamodels to predict and evaluate failure with a confidence above 95 % in initial service conditions of high-performance cylindrical vessels manufactured in composites by Roll Wrapping technology. Currently, there is no specific testing standardization for this type of vessel and to fill this gap probabilistic numerical models were developed, performed by the Finite Element Method, fed with the material characteristics obtained experimentally by 2D digital image correlation from flat specimens. From the initial numerical model, a surrogate metamodel was generated by stochastic approximations. Once the metamodels were obtained by robust engineering, an experimental ring-ring tensile test was developed under service conditions and deformations were measured by high-precision 3D digital image correlation. Parametric and robust tests showed that the results of the metamodel did not show statistically significant differences, with errors in the rupture part of less than 2 % with respect to the results obtained in the test, being proposed as a basis for new test procedures.

## Nomenclature

*DIC*: Digital Image Correlation
*DLT*: Direct Linear Transformation
*FEM*: Finite Element Method
*FI*: Failure Index
*FRP*: Fiber-Reinforced Polymers
*GoF*: Goodness of Fit
*MIG*: Mean Intensity Gradient
*PCE*: Polynomial Chaos Expansion
*PDF*: Probability Density Functions
*LOO*: Leave-One-Out
*RBDO*: Reliability Based-Design Optimization
*RHTT*: Ring Hoop Tensile Test
*ROI*: Region Of Interest
*RW*: Roll Wrapping

## 1 Introduction

Composite materials have become a reality, especially in sectors where the material used must be both lightweight and resistant, such as aerospace, shipbuilding, etc. The most common configuration is Fiber-Reinforced Polymers (FRP) [1,2], which is generated from a matrix, generally, epoxy, reinforced with a fiber whose main characteristic is a high mechanical resistance, such as carbon fiber (CFRP) or glass fiber (GFRP) [3,4]. The final properties of this composite will be a combination of the properties of the fibers and the matrix as a function of their volume fraction, in combination with manufacturing parameters and configurations such as fiber orientation, reinforcement direction, curing time, etc. [5].

There is a problem of uncertainty in defining the properties of composite materials or FRPs [6]. When characterizing them, a great disparity of results appears, due to the high number of parameters and great variability of them. It implies a very wide range of properties, compared to a very specific value as can occur in more conventional materials [[7], [8], [9]]. This is one of the reasons why traditional based-on deterministic simulation design methods are not the most suitable as they do not reflect this uncertainty. Stochastic methods are able to incorporate the uncertainty of the material factor, where they have shown to yield better results [10,11]. The precise definition of the material requires a large number of characterization tests whose objective is to define the behavior of the material through the Probability Density Functions (PDF) of the studied variable, which will be used to feed the numerical methods using the Finite Element Method (FEM) [[12], [13], [14], [15], [16]]. This method consists of discretization by means of nodes of a continuous medium allowing the simulation of the physical behaviors of different models. The application of numerical methods in combination with the different PDF will allow to obtain the failure probability of the system, thus achieving more reliable designs and avoiding the use of such a wide range of properties, hence the importance of a correct adjustment of the distribution. The FEM is a very useful tool that allows the integration of the probabilistic distribution of the variables of this type of material with more advanced engineering design tools such as robust engineering [14,17,18], the most suitable for design for materials being the one known as Reliability Based-Design Optimization (RBDO) [[19], [20], [21]]. This integration makes it possible to move from a deterministic FEM to stochastic FEM [[22], [23], [24]], which is a numerical model that allows the calculation of mechanical stresses and strains under real stresses, providing a complete study of results and considering the uncertainty that affects the input variables [25]. The problem is the high computational cost required for this operation. This problem can be solved with the use of the so-called surrogate models, where a high number of executions can be performed with a much lower computational cost without loss of accuracy [26,27]. In turn, surrogate methods, by making it possible to run multiple variations within the PDF of each feature, allow to evaluate the incidence/influence of each variable, selecting the variables that most influence the design in order to reduce the computational burden [24,28].

On the other hand, composite materials based on a polymeric matrix in combination with fibers are anisotropic materials, which do not have the same characteristics in all directions; therefore, local measurement techniques are not always valid and full-field techniques are required. The Digital Image Correlation (DIC) technique is well established in this regard, both in its 2D and 3D modes [[29], [30], [31], [32], [33], [34], [35], [36], [37], [38], [39]].

One of the most common manufacturing methods to obtain tubular geometries with composite materials is the manufacturing technique known as Roll Wrapping (RW) [40]. The testing and characterization methods for this manufacturing process have not been as widely developed compared to other types of composites and there are not global quality standards for the commissioning and failure prediction of this type of geometry [[41], [42], [43]].

The RW depends on several parameters which affect the final behavior of the product, such as winding pressure, curing time, number of layers, etc. A test protocol, Ring Hoop Tensile Test (RHTT), will be used based on digital image correlation that has been designed for this purpose as a validation and testing method. In addition, all characterization will be performed on pseudo-flat specimens cut from an RW shaped tube. The results will be statistically analyzed with a probabilistic approach oriented to the study of advanced materials and manufacturing methods.

Due to the very specific behavior of high-performance composite tanks, it is necessary to use advanced testing protocols such as RHTT [44,45]. This test is designed/oriented to calculate geometrically the mechanical behavior of pipes and tubes [46] and can also be applied to cylindrical vessels, since in this type of vessels the most critical load is usually radial, as in pipes. Taking all this into account, this work has employed a combination of DIC and stochastic simulation to improve predictive models of failure in high performance composite tanks.

Based on the indicated subjects, the authors want to know if it is possible to find a test that obtains a result closer to the in-service reliability of roll-wrapped tanks by combining digital image correlation and metamodels generated using probabilistic FEM. For this purpose, this work proposes a methodology that integrates various cutting-edge techniques in modern design. It begins with a comprehensive characterization of materials based on DIC, correlates this technique with numerical models for validation and refinement, and proceeds to develop a probabilistic model using stochastic methods to analyze the behavior of pressure tanks made of composite materials.

## 2 Materials and methods

### 2.1 Materials

The material used in this work is a Carbon Fiber Reinforce Polymer (CFRP) with epoxy resin matrix CR82® and 3 K carbon fiber 200 gr/m^2^ Twill weave 2 × 2 (twill) made by fiber Toray® T300. The used composite material has a total of 10 layers with ±45° orientations. The properties of the matrix and the fiber are outlined in Table 1, according to manufacturers datasheets.

**Table 1:** Mechanical properties of Toray ® T300 fiber and CR82® resin.

Resin	Viscosity	18,000–25,000 cps
	Glass Transition	130–140 °C
	Tensile Strength	50–70 MPa
	Elongation	4–5 %
Fiber	Density	1.78–1.82 g/cm^3^
	Tensile Strength	≥4000 MPa
	Elongation at break	≥1.5 %
	Young's Modulus	230–260 GPa

The cameras used for image acquisition are two identical bodies model Canon 700D DSLR cameras equipped with a 60 mm macro-lens. These cameras have a 22.3 mm × 14.9 mm APS-C CMOS sensor (pixel size of 4.3 μm). The image obtained is 5184 px x 3456 px.

All tests were carried out on a Servosis ME-405/50/5 tensile testing machine with a maximum load of 500 kN, with a TC50 Kn REP transducer as load cell.

### 2.2 Methods

**Fig. 1:**
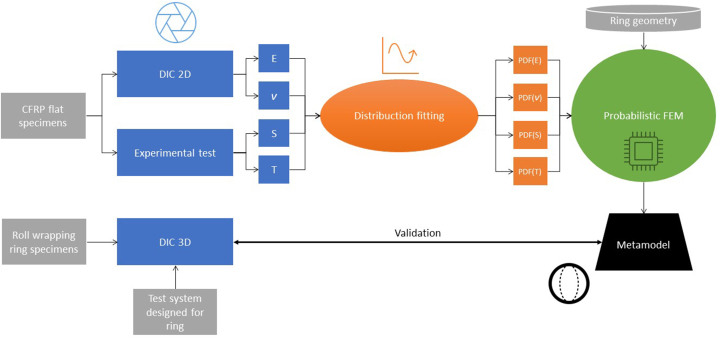
Workflow followed in the methodology applied.

**Fig. 2:**
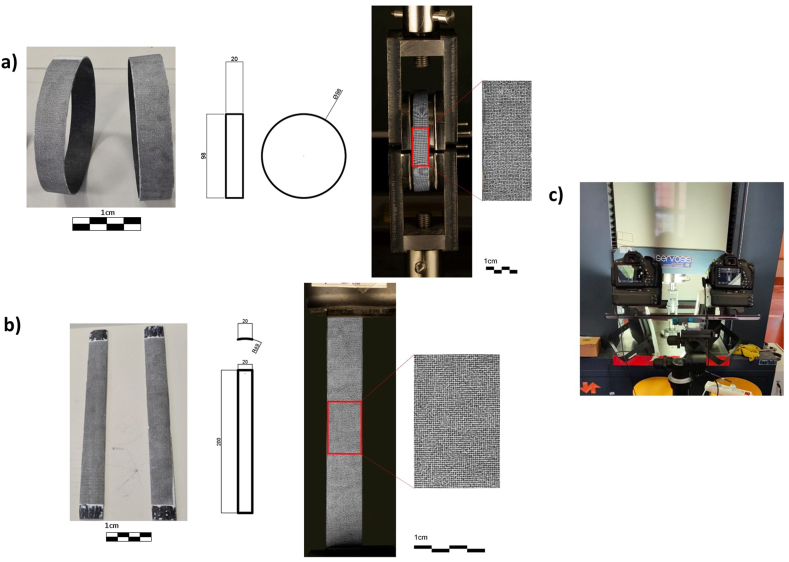
Dimensions and Speckle pattern: a) longitudinal specimen and b) ring specimen and c) Test setup.

The process has been specifically designed to evaluate the results of the surrogated metamodels and to compare them with the experimental results with the experimental results where the 3D DIC was implemented (Fig. 2). In order to include an accurate characterization of the final metamodels and to take into account the repeatability of the tests, different tensile tests on flat specimens have been performed by applying 2D DIC to generate probabilistic distributions of the measured variable. The main objective is to determine the main mechanical properties of the material by combining the different techniques. With DIC, Young's modulus (*E*) and Poisson's coefficient (*ν*) will be obtained. In a direct way, the Tensile Strength (*T*) will be obtained, which is necessary to determine the failure criterion, and, finally, the transversal elastic modulus (*S*) will also be obtained. With these initial data and their corresponding distributions, an initial numerical model will be fed through FEM. Taking into account the probability distribution functions PDFs of each property, multiple runs will be reiteratively performed by applying metamodeling and in this way a failure probability of this model will be obtained. The model will simulate the RHTT test on rings. This test has also been experimentally performed in laboratory and 3D DIC has been applied on it in order to have a complete field analysis of displacements and deformations, which will be directly compared with the FEM model. In this way, it will be possible to observe and analyze the probability of failure of the real specimens versus the numerical models, always supported with DIC methodology to be able to observe the theoretical-practical differences that appear (Fig. 1).

### 2.3 Tests set up

The specimens used for tensile tests are flat rectangular specimens with dimensions of 200 mm × 20 mm and a thickness of 1 mm. 20 specimens have been produced for this purpose. These specimens (Fig. 2) have been obtained by cutting them longitudinally from a 98 mm inner diameter tube. Due to the width/diameter ratio obtained, being 10–49 mm, it is considered that the curvature is practically negligible. The hoops have been obtained from transversal cuts on the same previous tube, 98 mm outside diameter and 1 mm thickness. The width of the hoops is 20 mm (Fig. 2).

For DIC analysis, as the reader can see in Fig. 2, a Speckle pattern has been placed on all specimens [47,48]. The main considerations to be taken into account are: i) randomness of the pattern; ii) circular dots and iii) coverage of about 40–70 % of the specimen surface to reduce the homogeneity of the specimen. The Mean Intensity Gradient (MIG) is the parameter used to ensure the quality of the pattern. Prior to applying the Speckle pattern, the specimen was coated with white matte spray paint to prevent glare that could distort the measurement. To ensure uniformity of illumination, two LED spotlights were placed so that they shine directly on the specimen. Both tests were performed with a specimen-to-camera distance of 1 m. In the case of DIC 3D the cameras are 260 mm apart.

Taking into account these specifications, a pattern based on circles with a diameter, *d*, between 3 and 5 pixels and a step between points has been designed. By applying a Gaussian random factor, the pattern is able to achieve a MIG of 56. The defined diameter is 0.324 mm and the step is 0.432 mm (Fig. 2).

The tensile test was carried out in accordance with ISO 527–4:2021 [49]. For the synchronization of the cameras with the measuring equipment, a Programmable Logic Controller (PLC) was used. This microcontroller will send a signal so that every second an image will be taken. Previous experiences testing CFRP allows to obtain 30 images per test using this trigger [50,51]. 2D DIC test allows obtaining the main mechanical properties of the material, such as Young's modulus, Poisson's ratio and failure strain.

For the 3D DIC test, according to ASTM D 2290–12 [52], tests can be performed on rings specimens in a similar way to a plane tensile test. Due to the manufacturing method, it is more consistent to perform tests on formed specimens than on a sheet material. This is partly due to the possible occurrence of prestresses and stresses due to the manufacturing process. For this reason, it is intended to carry out a campaign of tests on circular specimens, due to their similarity to the final product [53,54]. 36 ring specimens have been analyzed.

**Fig. 3:**
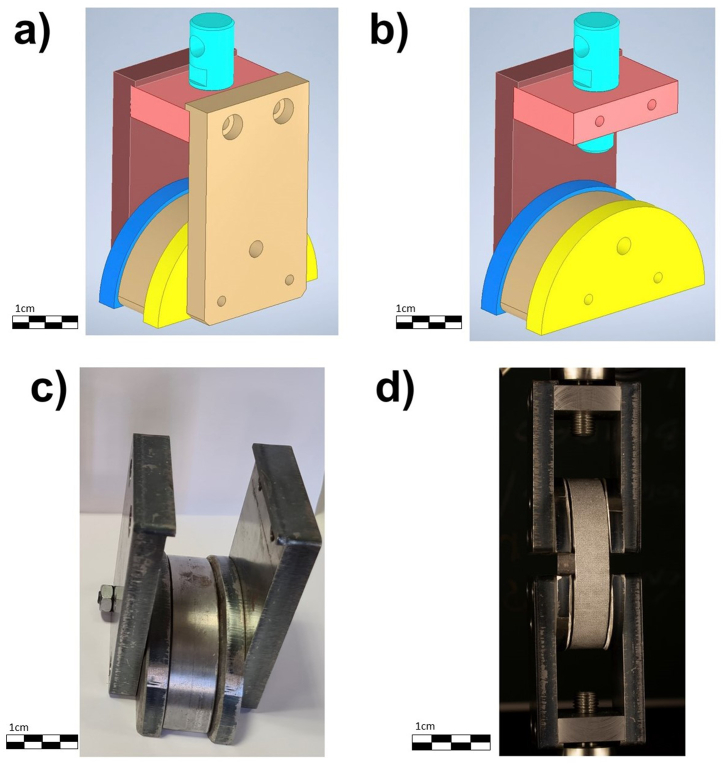
Ad hoc system to hold the 3D specimens: a) design of the system in Autodesk® Inventor; b) design of the system opened in Autodesk ® Inventor; c) real system manufactured and d) complete System.

In order to carry out these tests and in view of the lack of standardization in this respect, an ad hoc system has been designed to hold the specimens (rings). The system has a modular character to facilitate the assembly of the specimens, as well as their fastening. It has a semicircular piece (or main crescent) where the inside of the ring will be placed (Brown part in Fig. 3). Two half-moon-shaped plates are placed on both sides of the specimen to ensure a correct seating and centering of the specimen and it avoids possible lateral displacements (yellow and blue part in Fig. 3). Finally, on the outside of the assembly, two flat plates will be placed to connect the specimen seat to the testing machine. These pieces work by shearing and in turn have housings for the screws and pins that ensure the stability of the assembly during the whole test (Brown and maroon parts in Fig. 3). The side end plate is threaded to reinforce the system and avoid possible openings due to stress. In the upper part there is a plate where the side plates will be screwed and serves as a connection to the machine by a threaded system. All the parts are solid, except for the holes made to guarantee the non-existence of movements, they are made by stainless steel. This guarantees that the efforts transmitted will be supported without any problem by the machine.

#### 2.3.1 DIC processing

The 2D DIC technique will be used to make displacement and strain measurements in order to obtain the mechanical properties of the material. This is a non-contact technique that permits full field measurements unlike a strain gauge.

The method has already been described in previous works where this technique has been applied [50,51]. The DIC method compares subsets, square region divisions of the image, in consecutive images. The displacements are defined by the difference of image centroids, resulting in a displacement vector *A*.

Sub-pixel accuracy is achieved thanks to Quintic B Spline function [55]. It is noteworthy to mention that the Inverse-Compositional Gauss-Newton (IC-GN) algorithm has been used for the solution to find the optimization of the problem. The Ncorr tool has been used, which is a free software in Matlab ® [56].

All this is applied on a Region Of Interest (ROI). The Reliability-Guided Digital Image Correlation algorithm has been employed to minimize error propagation.

Once the deformations, *ε*, have been calculated, knowing at all times the force applied by the machine, and therefore the stress, *σ*, the Young's modulus, *E*, can be obtained. These calculations have been performed on the elastic range of the material. For this purpose, Hooke's law is applied (Equation (1)).

(1) $$E=\frac{\sigma }{\varepsilon }$$

To use 3D DIC, the principle of stereo view is required, so a minimum of 2 cameras is needed. In contrast to 2D, since the cameras are not perpendicular to the data acquisition, it is necessary to calculate the internal parameters of the camera as well as an orientation.

The relative orientation of the cameras was carried out using the Direct Linear Transformation (DLT). The use of DLT allows the image coordinates (*x*_*p*_, *y*_*p*_) to be related to the object coordinates (*X′*, *Y′*, *Z′*). In order to minimize the uncertainty in the depth axis, a cylindrical calibration target with points was used [50,51].

Where *x*_*p*_ and *y*_*p*_ are the coordinates of each point in the image and *L*_*1*_ – *L*_*11*_ are the mathematical parameters of the DLT.

Once the internal parameters of the camera were solved, the relative orientation of the cameras is performed using a cylinder pattern and a calibration plate [51]. For this, the following steps are followed: i) matching between the simultaneous images taken by the different cameras; ii) matching between successive stereo images, being the same as the one used in 2D DIC. After that, the DLT transformation has been applied to obtain the displacements of the subsets using the least squares approximation.

Thanks to the use of DLT it is possible to detect movements in depth, out-of-plane (*Z*). As a result, a full-field 3D point cloud of the specimen is obtained by calculating displacements and deformations using 2D DIC approximations.

#### 2.3.2 Numerical model

Two FEM models have been developed in order to make a direct comparison with respect to the results obtained with DIC 3D. These numerical models consist of the recreation of the same tests introducing all the uncertainties of the material.

• A 2D FEM model based on the plane tensile test.
• A 3D FEM model based on the RHTT test.

The plane tensile test consists of a single model corresponding to the specimen, with a total of 1350 nodes with SC8R element type distributed in such a way that the geometry of the specimen is similar to the real one with a total of 10 layers. Each layer has its corresponding mechanical properties. In order to simulate the tensile test, an encastre has been placed on the lower face, preventing any movement and rotation as it occurs with the lower jaw of the machine. To simulate the tensile test, a displacement type boundary condition has been placed on the upper face, this being the mean rupture displacement obtained from the tests.

**Fig. 4:**
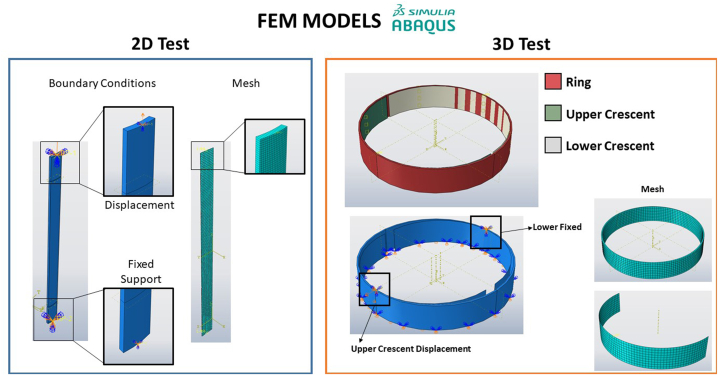
Different FEM models developed in 2D and 3D.

The RHTT test consists of 3 parts. Two rigid crescents, creating only the surface since the rest of the piece is not necessary and does not affect the model, achieving a significant reduction in computational cost. These pieces are equal, with a total of 675 nodes of type R3D4. The ring has been made with the same dimensions as the specimens to be tested. In this part there are 1368 nodes of type SC8R, distributed in 10 layers as in the previous model. In the same way as in the 2D model, an attempt has been made to simulate the RHTT test as accurately as possible. In this way, a Penalty type contact between the tools and the specimen has been configured. The movement system of the fixtures is exactly the same as for the 2D tests, so the lower fixture has been left fixed with an embedded type boundary condition, and the upper fixture has been configured to have a vertical displacement equal to the mean hoop breakage displacement. The models configuration are shown in Fig. 4. All models were made in Abaqus®, after that, metamodels were carried out in Matlab® using the free library UQLab ®.

The convergence study that has been carried out has taken into account the following aspects: i) size and complexity of the models studied, ii) the repetitiveness of the calculations for the preparation of metamodels and iii) the power of the available computers. Taking this into account, absolute convergence was not achieved, but the mesh size chosen does not significantly affect the results obtained, obtaining a feasible balance between the results obtained and the computational cost required.

#### 2.3.3 Surrogate metamodels

The uncertainty of the model is intrinsically controlled, being considered as another parameter within the method to generate the final results, which have been compared with the experimental results of the calibrated DIC.

The surrogate models allow an analysis to be made from multiple variations in properties to subsequently study the final performance without a significant loss of accuracy, which is why there is a great synergy between these methods and the composite materials [50,51].

To evaluate the PDF distribution that best fits the results obtained for each variable, three different tests will be used: Chi-Square (Chi), Kolmogorov-Smirnov (KS) and the Anderson-Darling test (AD) [57]. These tests will allow to estimate the PDF distribution that best fits the data. In addition, probability plots will be used to confirm adequate fit.

Once the mechanical properties have been statistically defined, a comparison criterion must be defined. For this purpose, the Tsai-Wu failure criterion is used (equation (2)) [58]. This criterion assumes that failure occurs when the Failure Index (FI) is greater than 1.

(2) $$FI=F_{1}\sigma_{1}+F_{2}\sigma_{2}+F_{3}\sigma_{3}+F_{11}\sigma_{1}^{2}+F_{22}\sigma_{2}^{2}+F_{33}\sigma_{3}^{2}+2F_{12}\sigma_{1}\sigma_{2}+2F_{23}\sigma_{2}\sigma_{3}+2F_{31}\sigma_{3}\sigma_{1}+F_{44}\sigma_{4}^{2}+F_{55}\sigma_{5}^{2}+F_{66}\sigma_{6}^{2}\leq 1$$

Where:

$$F_{1}=\frac{1}{X_{T}-\frac{1}{X_{C}}}\;F_{2}=\frac{1}{Y_{T}-\frac{1}{Y_{C}}}\;F_{3}=\frac{1}{Z_{T}-\frac{1}{Z_{C}}}$$

$$F_{11}=\frac{1}{X_{T}X_{C}}\;F_{22}=\frac{1}{Y_{T}Y_{C}}\;F_{33}=\frac{1}{Z_{T}Z_{C}}$$

$$F_{44}=\frac{1}{S_{yz}^{2}}\;F_{55}=\frac{1}{S_{zx}^{2}}\;F_{66}=\frac{1}{S_{xy}^{2}}$$

$$F_{12}=\left(-\frac{1}{2}\right)\sqrt{F_{11}F_{22}}\;F_{23}=\left(-\frac{1}{2}\right)\sqrt{F_{22}F_{33}}\;F_{31}=\left(-\frac{1}{2}\right)\sqrt{F_{33}F_{11}}$$

Where:

$\sigma_{n}\equiv$ Uniaxial tension in the different directions.

$X_{T}\equiv$ Tensile load.

$X_{C}\equiv$ Compression load.

$Y_{T}\equiv$ Transversal tensile load, in the *Y* direction.

$Z_{T}\equiv$ Transversal tensile load, in the *Z* direction.

$Y_{C}\equiv$ Transversal compression load, in the *Y* direction.

$Z_{C}\equiv$ Transversal compression load, in the *Z* direction.

$S_{xy};S_{yz};S_{zx}\equiv$ Shear transversal load.

Thus, the mean failure displacement in the tested specimens was taken and the Tsai Wu criterion [58] was implemented to define whether it has reached failure or not. This parameter has been determined because in the initial FEM model is the one that determines all the behavior. Thus, the rupture criterion is 1 with the final displacement. In order to compare all the statistical analysis, RBDO Analysis has been used (Equation (3)) [59]. This makes it possible to determine the probability of failure of the system:

(3) $${\;P}_{fk}=\int_{G_{k}\left(d,X\right)<0}f_{X}\left(X\right)dX$$

Where.

• $P_{fk}$ is the failure probability.
• $G_{k}$ are the constrictions.
• $f_{X}\left(X\right)$ is the probability density function (PDF) of the random vector $X\in R^{m}$ and the design vector $d\in R^{n}$.

However, the solution to this equation is not trivial and requires the use of approximation methods to calculate this value. For this work, it has been decided to use the Monte Carlo method for approximating this value [60,61]. This method requires the use of thousands of simulations to obtain results with confidence, demanding high computational costs [41,62,63]. In order to solve this problem, a metamodeling strategy has been used, in this case the Polynomial Chaos Expansion (PCE) method [[64], [65], [66]].

This method is a stochastic approximation of a system behavior through the spectral representation of random variables using a set of multivariate polynomials. The numerical simulation is assumed to represent a finite variance model, f(x) whose input x is a random vector of independence and constrained variables $X\in R^{m}$. This independence of inputs allows the definition of polynomials as the tensorization of univariate polynomials with respect to the marginal PDFs via equation (4):

(4) $$Y\approx f\left(x\right)=\sum_{\alpha \in A}\gamma_{a}+\varphi_{a}\left(x\right)$$

Where.

• α is the multi-index $\alpha =\left\{\alpha_{1},\cdots ,\alpha_{M}\right\}$.
• $A\subset N^{M}$ is the index set for the multivariate orthonormal polynomial.
• $\gamma_{a}$ are the deterministic coefficients to be computed.
• $\varphi_{a}\left(x\right)$ are the multivariate orthonormal polynomials.

For the estimation of coefficients (equation (5)) the least squares approximation has been used, posing a minimization problem between the vector of random inputs (X) and the response model (Y):.

(5) $$\varphi_{a}=\mathrm{argmin}\frac{1}{N}\sum_{i=1}^{N}{\left[\gamma^{\left(i\right)}-\sum_{\alpha \in A}\gamma_{a}\varphi_{a}\left(x^{\left(i\right)}\right)\right]}^{2}$$

To reach an overfitting situation with a large number of input dimensions, the adaptive sparse PCE based on minimum angle regression is used [67]. This type of procedure allows the resolution of engineering problems with a high number of dimensions. To guarantee the quality of the surrogated model, a version of the Leave-One-Out error (LOO) has been used (equation (6)) [68]. This error offers a good compromise between fair error estimation and affordable computational cost.

(6) $${LOO}_{error}=\frac{1}{N}{\sum_{i=1}^{N}\left(\frac{Y\left(X^{\left(i\right)}-f^{PCE}\left(X^{\left(i\right)}\right)\right)}{1-h_{i}}\right)}^{2}\;{LOO}_{error}^{*}={LOO}_{error}\cdot {\left(1-\frac{cardA}{N}\right)}^{-1}\left(1+tr{\left(\varphi^{T}\varphi \right)}^{-1}\right)$$

Where:

$Y\left(X^{\left(i\right)}\right)$ is the computational model.

$f^{PCE}\left(X^{\left(i\right)}\right)$ is the surrogated model of the specific Design of Experiment (DoE) with N samples.

$h_{i}$ is the ith diagonal term of the matrix $A{\left(A^{T}A\right)}^{-1}$, where A is the experimental matrix.

cardA is the number of terms in the truncate series and φ is shown in equation (7).

(7) $$\varphi =\left\{\varphi_{ij}=\varphi_{j}\left(X^{\left(i\right)}\right),i=1,\cdots ,N;j=1,\cdots ,cardA\right\}$$

### 2.4 Comparison between FEM and DIC

In order to validate the order of scale of these methodologies, a parametric and non-parametric statistic tests will be applied to compare the surrogated metamodel with respect the initial FEM model and also with respect the results of the experimental 3D DIC test. For this purpose, the nodes and points of the ROI in both 2D and 3D have been selected. A rotation and displacement have been performed, so that both models are located on the same axis with the same coordinate system. Thanks to this rotation and adjustment of axes, the difference between each node and point can be calculated in true magnitude. In this way, the displacements and deformations of both models can be directly compared.

## 3 Results

### 3.1 Mechanical properties of the material

First, mechanical properties were calculated from tensile tests and 2D DIC. In order to avoid perspective distortions, the camera has been placed orthogonal to the specimen, ensuring this perpendicularity with an inclinometer and a micrometric ball joint. To calculate both transverse and longitudinal displacements and deformations, virtual strain gauges have been placed on the processed specimens. Specifically, a ROI as large as possible has been selected avoiding conflicting points or shadows; 4 longitudinal extensometers with a uniform spacing of 10 mm and 4 transverse extensometers with a uniform spacing of 30 mm were used. It should be noted that, for both 2D and 3D DIC, an accuracy of 0.7 μm was achieved. This accuracy was achieved thanks to the fact that the preprocessing is the same for both techniques.

A total of 20 longitudinal planar specimens have been analyzed, with their corresponding values of Tensile Strength (*T*). Taking on account the extensometers, a total of 80 available results of Young's modulus (*E*) and Poisson's Coefficient (*ν*). The results of the transversal elastic modulus (*G*) tests have also been analyzed (Table 2).

**Table 2:** Statistical values of the mechanical properties obtained from the tests.

Variable	Mean	SE Mean	St.Dev.	Coef. Var.	Median	IQR	Skewness	Kurtosis
*E*	71.603	596	4766	6.66	71655	5007	0.21	2.22
*T*	810.732	9.35	74.79	9.23	817.30	73.02	−0.62	0.21
*ν*	0.01731	0.0196	0.1567	90.53	0.1240	0.1436	2.58	8.80
*G*	30.992	2.11	9.20	29.68	34.12	16.11	−0.45	−1.07

**Fig. 5:**
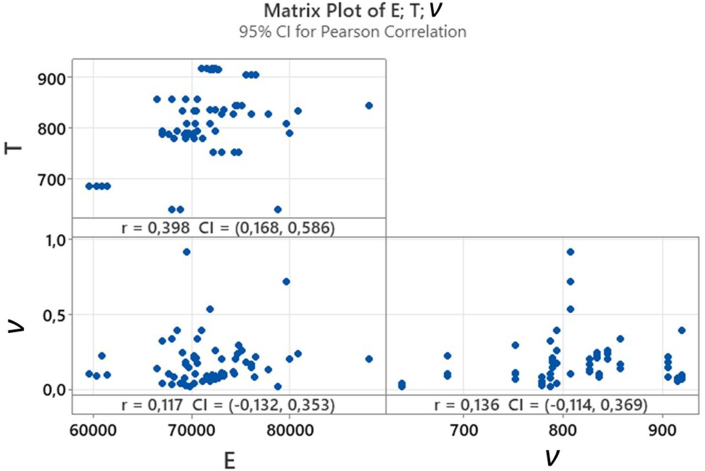
Matrix plot and *R-*Pearson correlation coefficient for *E*, *T* and *ν*.

A correlation study has been carried out between the variables obtained from the 2D DIC, which are the Young's Modulus (*E*), Poisson's Coefficient (*ν*) and Tensile Strength (*T*). A direct comparison has been made and the calculation of the correlation comes through a Pearson coefficient [69,70]. The aim of this comparison is to validate the necessity of surrogated models and the independence of study of the different variables. Results are shown in Fig. 5.

**Fig. 6:**
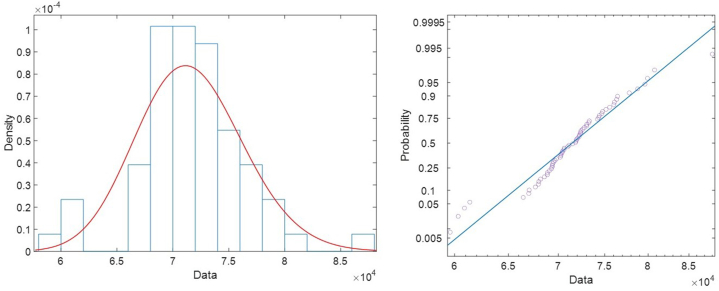
Best fit (lognormal) for *E* (left) and probability plot for lognormal distribution (right).

**Fig. 7:**
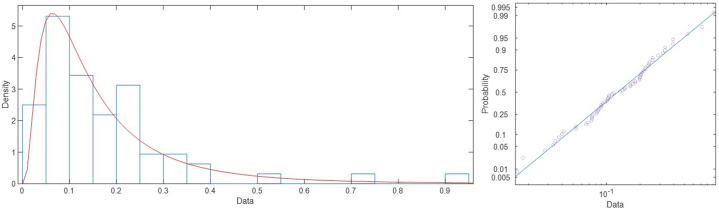
Best fit (lognormal) for *ν* (left) and probability plot for lognormal distribution (right).

**Fig. 8:**
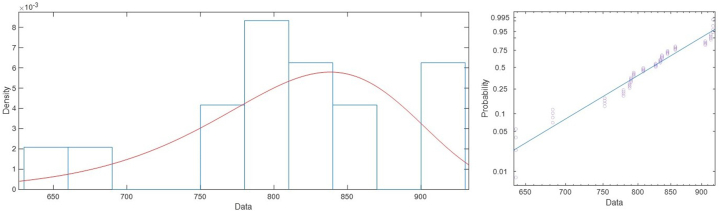
Best fit (Weibull) for *T* (left) and probability plot for Weibull distribution (right).

Given the variability of the results and to integrate the uncertainty within the process avoiding a classical deterministic approach, the study of Probabilistic Density Functions has been used. The probability of applying a Normal (N), Log-Normal (LN), Weibull (W) and Gamma (G) functions was studied. Subsequently, different Goodness of Fit (GoF) tests were used to validate the selection of these functions. Specifically, the Chi-Square (Chi), Kolmogorov-Smirnov (KS) and Anderson-Darling (AD) tests were used (Table 3). The results obtained show that *E* follows Log-Normal distributions while *ν, T* and G Weibull distributions. The mean of the distributions and its standard error is also used as a criterion to assess the differences between the fitted distributions (Table 4). The best fit plotted over the histogram and the probability plot for the distribution is shown in Fig. 6, Fig. 7, Fig. 8. As can be seen, all the tests done allows to consider that there is the best fit in the PDF distributions. This reduces the risk of a-priori error conditioning the final simulation results.

**Table 3:** Goodness of Fit (GoF) of the parameters obtained. A value of 0 indicates that the PDF is acceptable and 1 that it should be rejected.

Parameter	N			LN			W			G		
	Chi	KS	AD	Chi	KS	AD	Chi	KS	AD	Chi	KS	AD
*E*	0	0	0	0	0	0	1	0	0	0	0	0
*ν*	1	0	1	1	0	0	0	0	0	0	0	0
*T*	1	0	0	1	1	0	1	0	0	1	0	0
*S*	0	0	0	0	0	0	0	0	0	0	0	0

**Table 4:** Mean and standard error for each fitted distribution.

Distribution	Mean	Standard Error	95 % Normal CI	
			Lower	Upper
*E*				
Normal	71602.7	591.063	70444.3	72761.2
Lognormal^a^	71603.2	593.179	70450.0	72775.3
Weibull	71151.8	760.284	69677.1	72657.6
Gamma	71602.7	591.350	70443.7	72761.7
*ν*				
Normal	0.173112	0.0194353	0.135019	0.211204
Lognormal^a^	0.175621	0.0207471	0.139322	0.221378
Weibull	0.174483	0.0173623	0.143566	0.212057
Gamma	0.173111	0.0165515	0.140670	0.205550
*T*				
Normal	810.732	9.27586	792.551	828.912
Lognormal	810.830	9.66633	792.104	829.999
Weibull^a^	810.822	9.30871	792.781	829.274
Gamma	810.732	9.50710	792.098	829.366
*G*				
Normal	30.9915	2.05363	26.9665	35.0166
Lognormal	31.1325	2.40881	26.7518	36.2304
Weibull^a^	31.1298	1.93140	27.5655	35.1551
Gamma	30.9915	2.21000	26.6599	35.3231

a The best fit based on Anderson-Darling.

### 3.2 Surrogate model and correlation results

A comparison was made between the failure probability of the specimens tested with the mean rupture displacement. Subsequently, the independent probability of each specimen with this mean displacement was calculated. If it breaks, it will have a value Tsai-Wu ≥ 1. As explained in section 2, a numerical FEM model was used to simulate the test performed. In a first instance, 100,000 runs have been performed obtaining the distributions of the Tsai-Wu value shown in Fig. 11. However, there is a very significant difference between the surrogated model and the experimentally obtained values.

**Fig. 9:**
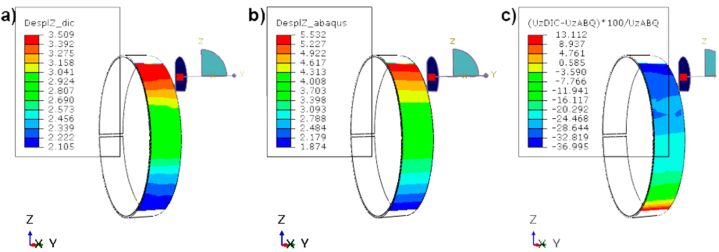
Results obtained by FEM and DIC and differences between methods: a) displacements obtained by DIC in mm; b) displacements obtained from FEM in mm and c) calculated error between FEM and DIC in %.

To determine the origin of the deviation, a comparison was made between the data obtained by 3D DIC with respect the data obtained by initial based-on FEM numerical model and also with respect the surrogated metamodel using parametric and non-parametric tests. The results have been compared in such a way that the difference between each node of the FEM model and the closest point to it in DIC has been recorded. In this way, the distribution of differences between DIC and FEM is observed in Fig. 9.

**Fig. 10:**
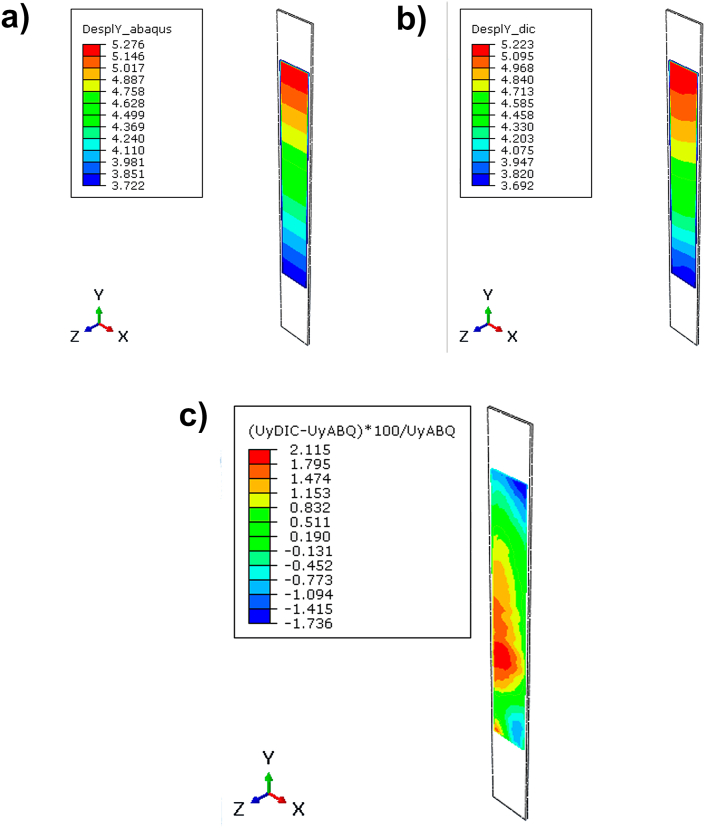
Differences between FEM and DIC: a) displacement result in FEM; b) displacement in DIC; and c) error between FEM and DIC.

As can be seen, the largest errors occur at the ends. This is due to the fact that the designed grips show a slight movement during the test because there is a large number of parts connected by pins and bolts and these can move along the application of the load. These large errors show that the comparison between methodologies is not valid, for this reason, a displacement measurement of these grips has been made, especially in the lower one through the different images during the test. Thanks to the data obtained by DIC 3D it has been possible to identify this problem, so that the verification of the FEM model using this technique has been possible. In this way, the result has been significantly adjusted. As validation, the same procedure has been carried out on the model and the 2D DIC, obtaining satisfactory results with errors lower than 3 % as shown in Fig. 10.

**Fig. 11:**
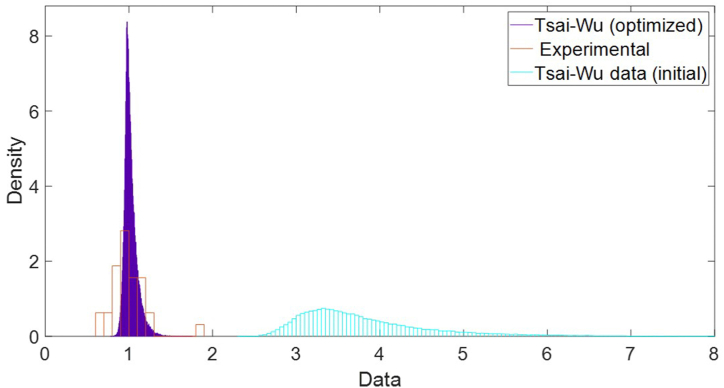
Histograms of the Tsai-Wu distributions (initial and optimized) with respect to the histogram of the experimental data obtained with DIC 3D.

Once the adjustment has been made, the metamodel has been run again adjusting these displacements detected in the DIC-FEM comparison. 100,000 runs have been performed again and the result obtained in this case is observed in Fig. 11.

**Fig. 12:**
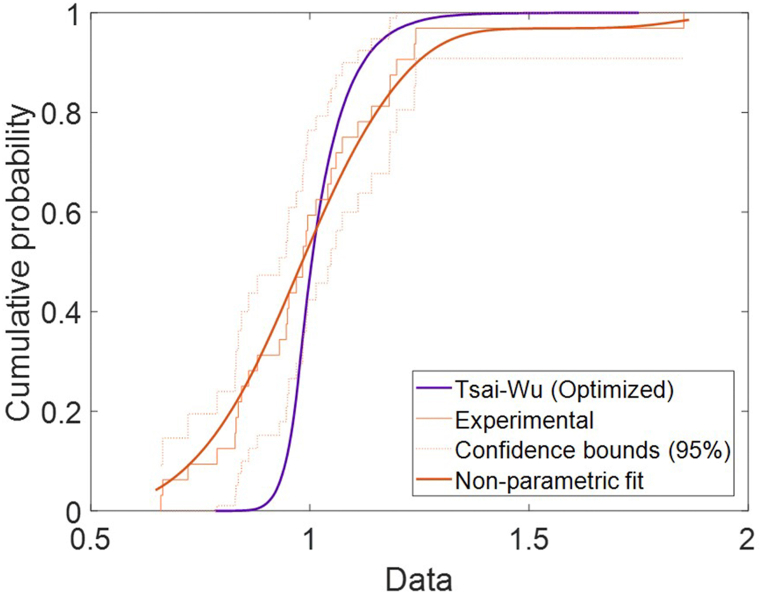
Cumulative distribution function (CDF) representation of Tsai Wu.

The Cumulative Density Function (CDF) was calculated for both the optimized Tsai-Wu model. Using the experimental data, a non-parametric probability distribution was fitted, and the 95 % confidence bands were calculated. As the reader can see (Fig. 12), the function of the optimized method falls within the bands starting at approximately 0.9. The corrected results are already remarkably similar to those of the optimized method (Table 4) to the values obtained directly to those of the real specimens.

Taking into account the non-normality of the data distributions evaluated using Kolmogorov-Smirnov and Anderson-Darling (Table 5), the nonparametric U-Man White test was conducted. Statistically significant differences were found between the experimental results with respect the first model (*p* = 0.000), also between the first metamodel and the corrected metamodel (*p* = 0.000), and between the real and optimized metamodel (*p* = 0.000). This shows that there are statistically significant differences in the medians (robust test) between the first model respect to the corrected model and relate to the experimental values.

**Table 5:** Comparison of quartiles on the data obtained by the tests, the first metamodel and the adjusted metamodel.

	Tests	1st Model	Error (%)	Corrected Model	Error (%)
Quartile 1	0.6596	2.3172	251.30	0.7836	18.80
Quartile 2	0.8437	3.2484	285.02	0.9718	15.18
Quartile 3	0.9835	3.6106	267.12	1.0042	2.10
Quartile 4	1.0739	4.1543	286.00	1.0554	1.72
IQR	0.3239	1.2989	401.01	0.2206	31.89
K–S	<0.000	<0.000	0	<0.000	0
A-D	0.032	0.001	96.88	0.001	96.88

However, when this test is applied to evaluate the difference between the medians of the test and the corrected metamodel, no statistically significant differences were detected in the median (*p* = .113). Nevertheless, the analysis of quartiles does show critical differences in the variability of the process but the experimental results present a greater variability than those provided by the corrected model (Table 5). The calculated error is the relative error with respect to the data obtained by the cumulative experimental data shown in Fig. 12.

#### 3.2.1 3D DIC calibration

To ensure the robustness of the results, it is important to clarify the calibration parameters obtained from the cameras and their calibration according to the 3D DIC technique, as shown in the following Table 6.

**Table 6:** 3D DIC cameras calibration parameters.

Parameter		Camera 1		Camera 2	
		Initial	Calibrated	Initial	Calibrated
**Principal Point (pixel)**	***x***_***p***_	2.6196 E3	2.6264 E3	2.6329 E3	2.6294 E3
	***y***_***p***_	1.8309 E3	1.8299 E3	1.8473 E3	1.8473 E3
**Radial** **Distortion**	***k***_***1***_	0	−1.861E-4	0	7.944 E−5
	***k***_***2***_	0	3.591 E−2	0	−3.763 E−3
	***k***_***3***_	0	−1.143	0	1.687 E−1
**Tangential** **Distortion**	***p***_***1***_	0	−2.078 E−5	0	−5.237 E−7
	***p***_***2***_	0	7.772 E−5	0	−7.245 E−5

## 4 Discussion

As it has been observed, the system is very sensitive to inaccuracies and any failure is very important when it comes to correctly defining this type of material In this sense, the literature shows that stochastic approaches are the best solutions [71,72]. For this reason, the DIC is not only a powerful tool for the determination of the mechanical properties of these, but it is also a very useful tool to observe and check what is happening, during the data collection and in a full field of the specimen [29,73], and what solutions can be provided.

**Fig. 13:**
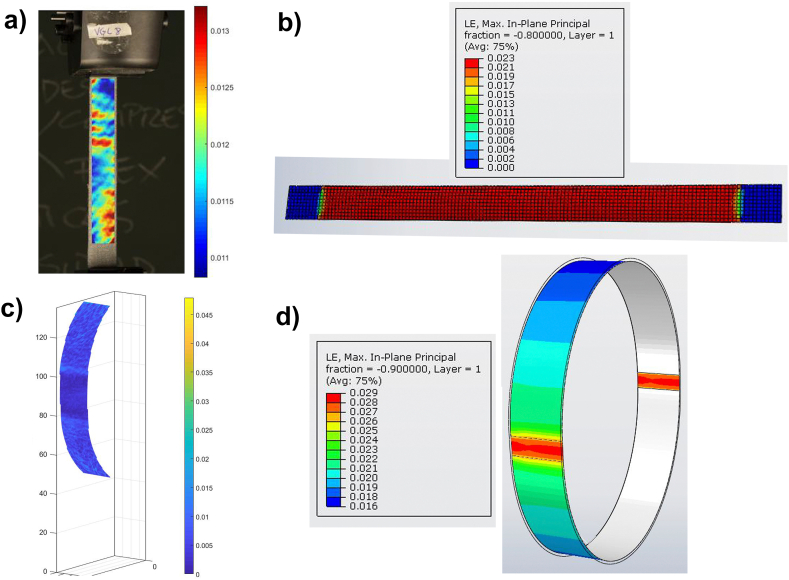
Comparison of the distribution of the deformations between real model measured by DIC and theorical FEM model: a) deformations recorded by DIC 2D; b) deformations on the 2D FEM model; c) deformations recorded by DIC 3D and d) deformations on the 3D FEM model.

The main discrepancies between tests and simulations are usually given by the anisotropic character of the material, in traditional materials, the results are very similar between the different techniques, focusing on the subset of the DIC technique [74,75]. The behavior of the material has a large variation depending on the direction of the stress, therefore, characterizing and replicating this behavior accurately is not always possible. Comparing the deformation map in both 2D and 3D, it is observed that with the DIC this large variation of deformations is recorded without any clear pattern, as shown in Fig. 13. The reached results are in agreement with the conclusions reached in other studies [76]. Due to the large variation of properties in anisotropic materials in different directions, applying a global DIC leads to a loss of accuracy in obtaining results. This causes differences between the theoretical model and the results obtained by the DIC, so it is necessary to adjust the model in order to obtain the true behavior.

For this reason, the use of direct geometric relationships applied on conventional materials on the RHTT are not feasible when the material is anisotropic and has a high rate of variation as in the case of high-performance deposits made of composite materials. The optimization of the metamodel allows generating a failure model that resembles the one extracted by testing. The centering of both distributions is similar but the surrogated model has a lower variability than the one obtained by physical testing. This fact may be due to the variability that occurs in the physical tests and to the anisotropy of the physical properties of the composite and possibly also to the error in the adjustments of distributions of *E*, *ν* and *T* parameters, obtained experimentally.

The application of destructive physical tests provides greater reliability, but the increase in costs and time is significant, making it impossible to use them more extensively. For this reason, metamodels make testing processes more efficient, saving costs, both economic and computational, and time. If it is possible to extend the number of destructive tests, metamodels can be complementary and can serve as a basis for the application of Industry 4.0 technologies, such as digital twins, or be compatible with learning algorithms oriented to artificial intelligence.

In the current literature there is no direct comparison between nodes simulated in FEM and results obtained by DIC directly and in full field. However, what is found is the comparison of properties obtained and simulated global behavior with a very small error [74,76]. In this work, it has been possible to obtain a full field comparison, being able to observe where these concrete differences between the theoretical FEM model and the experimental results obtained by the DIC are found since having anisotropic materials, making a global comparison would have an intrinsically high error.

## 5 Conclusions

Testing protocols of high-performance tanks built in composite materials require powerful tools capable of giving an accurate and full-field response of displacements and deformations. Therefore, it has been demonstrated that the DIC technique is a useful and adequate technology to record these behaviors, obtaining errors, especially in flat behaviors, lower than 2 % between the real model and the theoretical model of behavior.

The theoretical behavior of this type of materials is difficult to predict due to the anisotropic characteristics of composite materials. In this sense, the feeding of subrogated models with the mechanical characterization of the materials with 2D DIC, performing a stochastic simulation, offers differences between the theoretical values and the real approximate values in the rupture zone, where it is more relevant. In this case, thanks to the DIC technique, it has been possible to obtain a more realistic theoretical simulation, so that the errors in the results are less than 2 % once the model has been corrected, compared to about 300 % for the first theoretical model. it has therefore been corroborated by a solid comparison with the experimental results, in which no statistically significant differences were found between the results based on metamodels and the experimental results. Thus, it is possible to establish models that serve as a basis for predicting failures with more than 95 % confidence when composite tanks are put into service. This would save costly and complex tank tests that would require high working pressures, long test times and the generation of waste and contamination.

Therefore, the workflow created in this work combines complete techniques such as 2D and 3D DIC with advanced stochastic numerical models, offering a possible answer to the demand for standardization of testing and prediction of mechanical behavior in high performance cylindrical vessels, giving predictions above 95 % reliability in terms of breakage.

## Additional information

No additional information is available for this paper.

## Data availability statement

Data included in article/supp. material/referenced in article.

## CRediT authorship contribution statement

**Javier Pisonero:** Writing – original draft, Methodology, Investigation, Formal analysis, Data curation, Conceptualization. **Manuel Rodríguez-Martín:** Writing – original draft, Validation, Methodology, Investigation, Formal analysis, Conceptualization. **Jose G. Fueyo:** Writing – review & editing, Validation, Formal analysis. **Diego González-Aguilera:** Writing – review & editing, Visualization, Supervision. **Roberto García-Martín:** Writing – review & editing, Visualization, Supervision, Resources, Funding acquisition.

## Declaration of competing interest

Authors declares no conflicts of interest
